# OSMlanduse a dataset of European Union land use at 10 m resolution derived from OpenStreetMap and Sentinel-2

**DOI:** 10.1038/s41597-025-04703-8

**Published:** 2025-05-06

**Authors:** Michael Schultz, Hao Li, Zhaoyan Wu, Daniel Wiell, Michael Auer, Zipf Alexander

**Affiliations:** 1 Geoinformatics of University of Heidelberg, Heidelberg, Germany; 2 Department of Geography, National University of Singapore, Singapore, Singapore; 3 Zwsoft Co. Ltd., Guangzhou, China; 4 United Nations Food and Agriculture Organization, Rome, Italy; 5 Heidelberg Institute for Geoinformation Technology, Heidelberg, Germany

**Keywords:** Geography, Environmental impact

## Abstract

Our map represents the first successful large-area fusion of OpenStreetMap and Copernicus data at a spatial resolution of 10 m or finer and can be applied globally. We addressed varying label noise and feature space quality, utilizing artificial intelligence and advanced computing. Our method relies solely on openly available data streams and methods, eliminating training data acquisition or the need for additional expert knowledge for such purpose. We extracted land use labels from OpenStreetMap and remote sensing data to create a contiguous land use map of the European Union as of March 2020. OpenStreetMap tags were translated into land use labels, directly mapping 61.8% of the Union’s area. These labels served as training data for a classification model, predicting land use in remaining areas. Country-specific deep learning convolutional neural networks and Sentinel-2 feature space composites of 2020 at 10 m resolution were employed. The overall map accuracy is 89%, with class-specific accuracies ranging from 77% to 99%. The data set is available for download from 10.11588/data/IUTCDN and visualization at https://osmlanduse.org.

## Background & Summary

Humans transformed most of the Earth’s terrestrial surface^1^. Spatial and temporal explicit accurate land use (LU) and land cover (LC) information^2^ is useful, to understand environmental dynamics and anthropogenic activities^3^. The creation of consistent large area LULC products benefitted most notably by the use of remote sensing, its proliferation through open data policies^4^ and artificial intelligence^5^. Currently the further accelerated use of such technology is primarily limited by the availability of sufficient thematically labelled data to improve training performance of artificial intelligence for classification tasks.

Many non-commercial LULC maps were produced through authoritative^6,7^ or academic^8,9^ efforts where unrestricted training data accessibility for anyone is absent by design. With the advent of open-access web-based digitalization within the first decade of the 21^st^ century, cost effective LULC originating from citizen science (CS) emerged^10,11^ tapping into a novel source of labels and training data based on open collaborative mapping platforms, most notably OpenStreetMap (OSM)^12^. OSM is a web based opportunistic collection of spatial explicit vector geometries enriched with thematic attributes collected by humans primarily through remote mapping or *in situ* data acquisition. Wherever OSM data is available and enriched with themed and up to date content, it can be sourced for LULC purposes, both spatial explicit and related to defined temporal periods. For instance, by translating its thematic content into a practical LULC class using a certain version of the respective OSM objects^13^. Although guidelines and some safeguards are provided by the OSM community, they can’t be enforced, anyone can contribute anything, as a result data quality varies largely. Additionally, the platform suffers particularly from incompleteness due to the nature of opportunistic human driven data input. OSM edits are based on certain local interest groups, and can be tied to certain events^14^ or to complement a targeted service or purpose^15^. Yet, despite these limitations, it is the largest and most successful open, free to use, non-commercial mapping project to date and used every day as reliable base map, routing device and information platform by an abundancy of commercial, governmental, and non-governmental institutions^16^.

Given the fragmented availability of OSM data and opportunistic availability of thematic content and depth, resulting LULC products are consequently incomplete in terms of spatial coverage. Such gaps in unlabelled LULC data can be addressed by performing basic classification of remote sensing data, using known areas as training data^17^. Related research has focused on identifying useful OSM tags and their geometries for representing LULC^18^ as well as leveraging machine learning to fill data gaps^19^. Label based deep learning (DL) classification of RS data became an alternative cost effective tool to produce LULC maps^20^.

Although previously mentioned studies demonstrated the suitability of OSM facilitation for LULC, a large area demonstration of these tools was missing but is provided through this work. Furthermore, our approach offers excessive amounts of training data that can suit DL methods. Both, OSM and remote sensing share omnipresence in time and space for most of the 21^st^ century offering transferability and scalability. Here we demonstrate the combination of OSM and remote sensing for a 10 m land use map for the countries of the European Union (EU). Our product follows the coordination of information on the environment (CORINE) LC (CLC) legend offering comparison, integration to national products and European products that are in conformity to these legend requirements. Our product may serve as a complementary map for CLC or individual product by design our method can be applied for anytime and anyplace where a certain amount of remote sensing data and OSM data is available.

## Methods

**Fig. 1:**
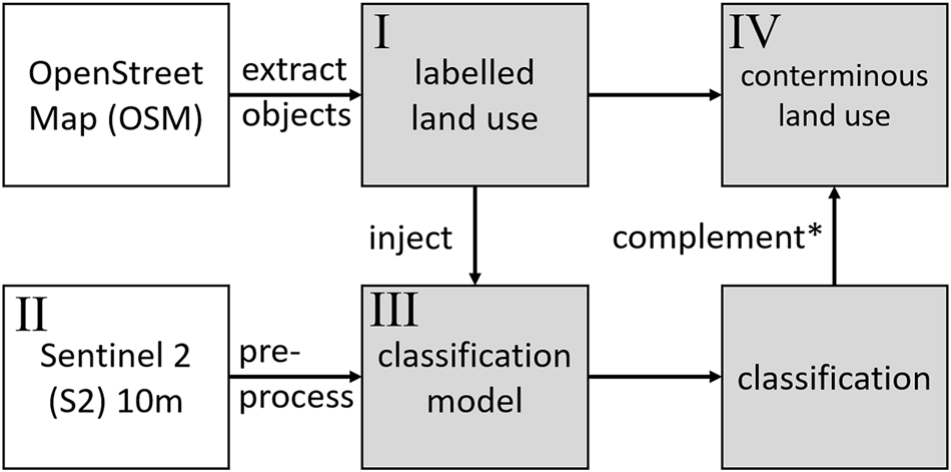
Research design for map production where roman letters I – IV show order and flow of events, grey boxes indicate data and models available within the paper’s repository; *classification was used when labelled land use was absent; OSM data can be retrieved through OSM API, pre-processed S2 data through FAO’s sepal.io.

By injecting known labels extracted from OSM into a Sentinel 2 best pixel feature space using deep learning, CORINE land use labels were predicted when absent thus creating a contiguous map. The study design was displayed in Fig. 1. First, OSM data was queried and translated to CLC labels^17^ using the OSM version as of March 2020. We retrieved OpenStreetMap (OSM) vector data [Ref.], ensuring compliance with the Open Database License (ODbL)^21^. Where available the data served directly as the LULC product at <1 m resolution (OSM vector geometry) and as training data labels for a deep learning-based classification^22^ using a Copernicus Sentinel 2 (S2) 10 m multi spectral feature space processed through Food and Agriculture Organization (FAO) sepal.io system. Subsequently the relevant aspects of the product design are presented.

Feature space was a best pixels medoid composite of Sentinel 2 bands red, green, blue (RGB) and near infrared (NIR) at 10 m of the past three years as of April 2020. Data pre-processing of the feature space was based on Landsat^23^ and adjusted for Sentinel 2 bands. First, clouds and cloud shadows were removed using fmask^24^. Second, for each pixel’s time series the medoid pixel was selected, pixels acquired during the growing season were preferred. Finally, the cloud screened best pixel mosaic composite was projected to EPSG:3035. Pre-processed Sentinel 2 composites can be created for any place and queried free of charge using the above-mentioned configuration through the FAO SEPAL platform sepal.io^25^.

Training data, labels for the supervised classification were extracted from OSM where certain tags were associated to 13 CLC classes described here^17^. OSM feature quality, abundancy, and the suitability of OSM to land use conversation varies by country^26^ and remote sensing feature spaces properties vary by landscape. We accommodated these spatial variations by an individual set of training data and feature space for each country rather than pooling them. Consequently, each country received its own set of training data and classification model.

Classification of remote sensing data feature space was performed through the labelled training data, which was injected into a non-parametric supervised residual convolutional neural network (ResNet), complemented by identity mapping^27^ For each country we used the same deep learning model architecture, but feature space and training data varied, thus resulting in an individual model for each country. Classification chunks were portioned by the footprint size of Sentinel 2 data. ResNet inherent residual blocks (RB) were used to alleviate the gradient fluxes of convolutional neural networks (CNN)s during training^28^. By solving the optimization degradation issue, such blocks were used to increase training accuracy. Here, ResNet with multiple RBs were selected as the base feature learning networks to learn the targeted countries discriminative features. To feed the learning model we selected for each Sentinel 2 tile and class category 5000 training samples randomly. Sampling based reduction of training data was performed to cope with overly abundant yet noisy training data. Training and validation ratio were 0.8 and epochs sealed at 200 iterations. We used Nesterov Adam optimizer to improve convergence performance of leaning rates. Respective, hyperparameters were set to β1 = 0.9, β2 = 0.999, learning rate and batch size were set to 0.001 and 64, respectively. Model inferencing of the trained ResNet was applied through a moving window where each pixel with its neighbour batches is classified into the unique LULC classes. The resulting classification was post processed by an additional sieve filter of 64 pixels. Finally, the classification and the original labels were merged while giving priority to the existing labels.

Our decision to train separate classification models per country stems from the varying completeness and likely slightly different tagging cultures of OpenStreetMap data across Europe, may differ at national borders. We used a CORINE-based legend to ensure overall harmonization, yet small discrepancies remain due to differing OSM tag usage. Moreover, although the final dataset is integrated at the European scale, we conducted both a pan-European accuracy assessment and additional per-country accuracy reporting to reflect each model’s distinct performance. We acknowledge that fully eliminating cross-border mismatches requires continued refinement of OSM tags and further post-processing, but we see this dataset as an important step toward large-area, openly sourced land-use products at high spatial resolution.

## Data Records

The dataset^29^ is provided as individual GeoTIFF files (one per EU country plus the UK) at 10 m spatial resolution. Each GeoTIFF encodes land-use class labels following the CORINE Land Cover (CLC) nomenclature^30^ and the specific class codes are listed in Table 1 of this manuscript. The data can be downloaded from 10.11588/data/IUTCDN, which hosts both the predicted land-use rasters and the accompanying metadata describing file format, spatial reference, class definitions and visualization are available at https://osmlanduse.org.

**Table 1:** Legend harmonization between OSM tags and Corine Land Cover (CLC) classes, level two legend.

CLC Class – level 1	CLC Class – level 2	CLC Class Name	Corresponding OSM tag
1	1.1	Urban fabric	residential
	1.2	Industrial, commercial and transport units	industrial, commercial, retail, harbour, port, railway, lock, marina
	1.3	Mine, dump and construction sites	quarry, construction, landfill, brownfield
	1.4	Artificial non-agricultural vegetated areas	stadium, recreation_ground, golf_course, sports_center, common, allotments, playground, pitch, village_green, cemetery, park, zoo, track, garden
2	2.1	Arable land	greenhose_horticulture, greenhouse, farmland, farm, farmyard
	2.2	Permanent crops	vineyard, orchard
	2.3	Pastures	meadow
3	3.1	Forests	forest, wood
	3.2	Shrub and/or herbaceous vegetation associations	grass, greenfield, scrub, heath, grassland
	3.3	Open spaces with little or no vegetation	fell, sand, scree, beach, mud, glacier, rock, cliff
4	4.1	Inland wetlands	march, wetland
	4.2	Coastal wetlands	salt_pond, tidal
5	5.0	Water bodies	water, riverbank, reservoir, basin, dock, canal, pond

## Technical Validation

Using an independent reference data set consisting of 4616 reference points an overall accuracy of 89% was achieved for the EU OSMlanduse product. Independently collected robust reference data revealed class accuracies ranged from 77% - 99%. The largest class confusion was found for Artificial surfaces (10) and Forest and seminatural areas (30). An expert-based detail investigation revealed that this error was mostly driven by the misclassification of “Artificial non-agricultural vegetated areas” (14) and “Shrub and/or herbaceous vegetation associations” (32), two spectrally very similar classes. The class “Artificial surface” was overestimated on the cost of “Forest and seminatural areas”. However, existing areas of class “Artificial surface” were mapped almost entirely correct with only minor errors of omission (97% producer accuracy).

Accuracy was calculated using remote sensing standard procedures^31^ consisting of sampling and response design as well as standard analysis measures^32^. The sampling design determined the distribution of the reference points across the data set. Sampling size population was calculated using standard random stratified sampling using formula 1 suggested in^33^ using a standard significance level (α = 0.95) and confidence interval (h = 0.05). For the extraction of the representation of reality, the response design, we used very high resolution (<1 m) remote sensing ranging from the years 2019 and 2020 provided by Bing Maps (Bing Aerial) and google maps (Satellite view) as well as an additionally 10 m auxiliary layer of Sentinel 2 RGB provided by Sinergise SentinelHub as of March 2020 accommodating a perspective of the reference point for the very moment in time of the product creation. At each reference point the land use was extracted and interpreted for a 10 m × 10 m box, the class that occupied the majority of the reference point was then assigned as representation of reality for the respective location and consequently compared against the map. Through analysis we determined the difference of the map and the reference data using the measures overall accuracy, producer’s accuracy or omission error expressing the underestimation of a class and user’s accuracy or commission error expressing overestimation of a class, were calculated, and reported in Table 2.

**Table 2:** Confusion matrix for a total of 4616 reference/validation points.

		Map			Σ	producer accuracy
		1 - Artificial surfaces	2 - Agricultural areas	3 - Forest and seminnatural areas		
Reference	1 - Artificial surfaces	1419	29	3	1451	0.977946
	2 - Agricultural areas	134	990	9	1133	0.873786
	3 - Forest and seminatural areas	283	43	1706	2032	0.839567
	Σ	1836	1062	1718		
	user accuracy	0.772876	0.932203	0.993015		0.891464

To ensure unbiased interpretation of samples, reference data was collected through a crowd sourcing campaign hosted by landsense.eu. The campaign was open for the public from October 2020 until March 2021. The campaign was stimulated by three dedicated mapathon events that were featured during these events namely, EU regions week on 14.10.2020, the Netzwerk Geoinformation der Metropolregion Rhein-Neckar (GeoNet.MRN) on 29.10.2020 and during a dedicated validation campaign hosted by European Spatial Data Research (EuroSDR) on 24.11.2020. A reference point made it into the reference data base if at least three different individuals agreed on a certain class without knowledge of one another’s decision or the respective map category. At least 60 interpreters participated in the validation effort performing at least 20k interpretations. Provided thematic depth varied, thus we aggregated classes to CLC level 1, wetland and water classes were disregarded.

Figure 4 provides a cross tabulation of existing labels and their prediction revealing both, model performance and training data noise. We used producer and user accuracy as vehicle to express these differences. Figure 4 offers insights on the prediction reliability for each class and each country, and highlights trends and notable examples. Some classes were comparatively well classified for most countries, such as “Water” (5), “Forests” (31) and “Urban fabric” (11) while others were characterized by consistently low performances across countries, namely “Mine, dump and construction sites” (13) and “Artificial non-agricultural vegetated areas” (14). Variation of model performances showed similar patterns across countries yet there is no country that specifically excels at all classes or a certain set of classes, top scorers differ from class to class.

In the figure, the accuracies of specific countries are highlighted in orange labels, emphasizing the extremes and country specific suitability’s of the method. Notably, Malta exhibits the lowest model performance across multiple classes, including urban fabric, arable land, forests, artificial non-agricultural areas, water, and shrubs. This observation is remarkable, as Malta’s performance is consistently poor across these categories, however other countries especially Germany, Romania and Austria perform exceptionally well.

**Fig. 2:**
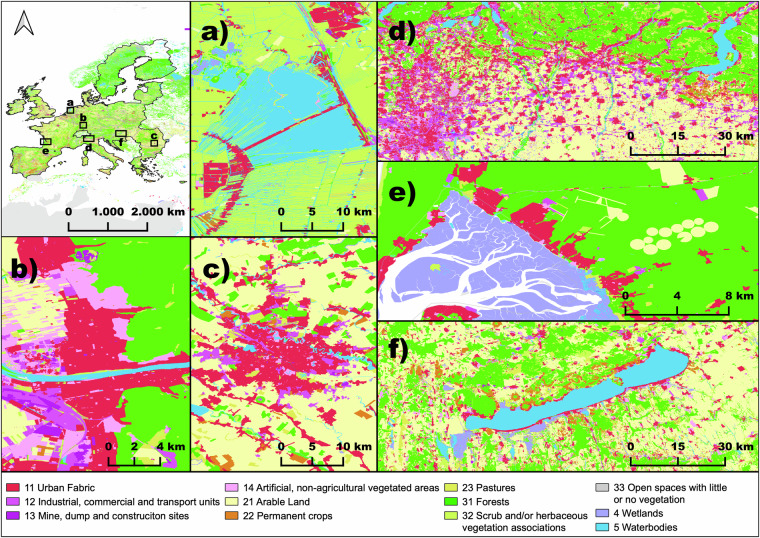
Map facets highlighting the OSMlanduse details and suitability for various landscapes (**a**) inland water in Utrecht province, (**b**) city of Heidelberg, (**c**) metropolitan area of Bucharest, (**d**) Po valley and Milan, (**e**) wetlands river estuary, close to Bordeaux (**f**) lake Balaton close to Siófok.

**Fig. 3:**
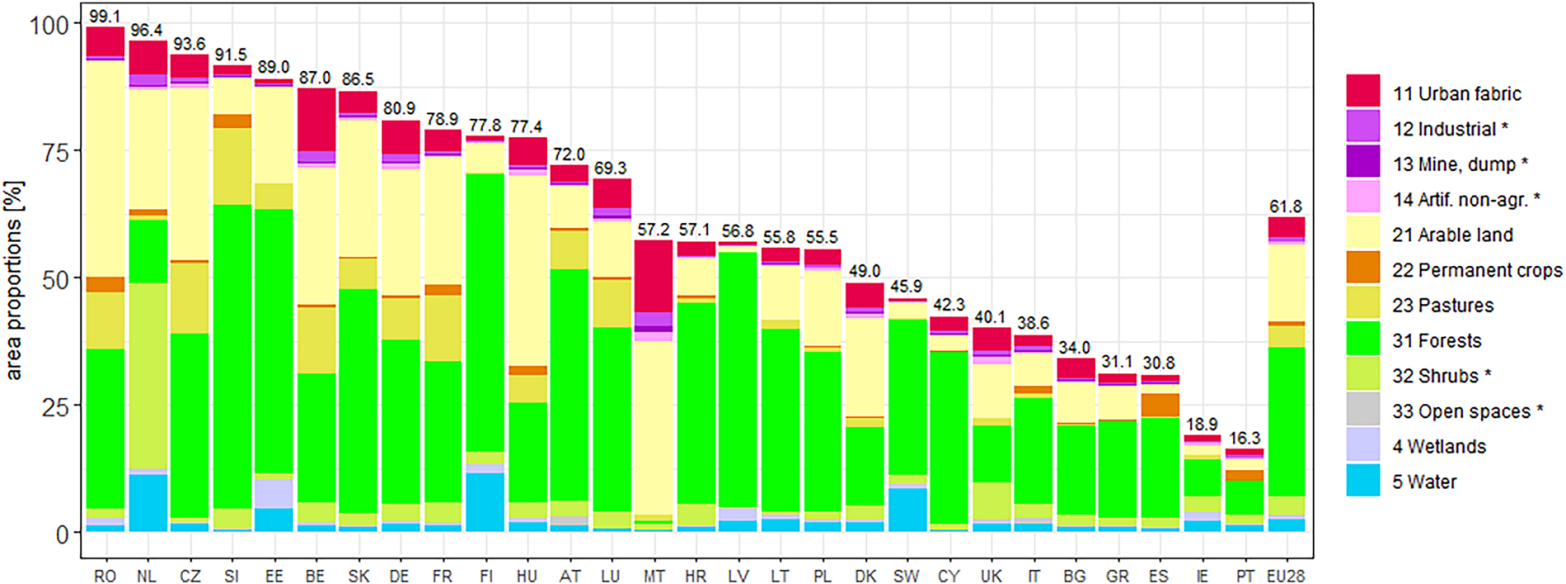
Availability of existing OSM labels used as training data and product, as a fraction of the country’s total area; EU28 = all countries combined relative proportions.

Figure 2 provided map examples of the OSMlanduse product as seen on osmlanduse.org. Here, areas that were directly converted from OSM to OSMlanduse are displayed as vector geometries <1 m resolution and areas filled through deep learning as 10 m raster grid. Figure 3 showed that approximately 61.8% of the entire study site was covered by relevant OSM data used as labels. In relation to their country size the label availability varied largely for the classified countries from 16.3% - 99.1% Within our data records we provided the OSMlanduse labels used as training data at 10 m resolution and the full product at the same resolution. The vector based OSMlanduse product can be extracted directly through any OSM suited application programming interface (api) using the key-value pair combinations provided in^17^ or shown in Table 2.

**Fig. 4:**
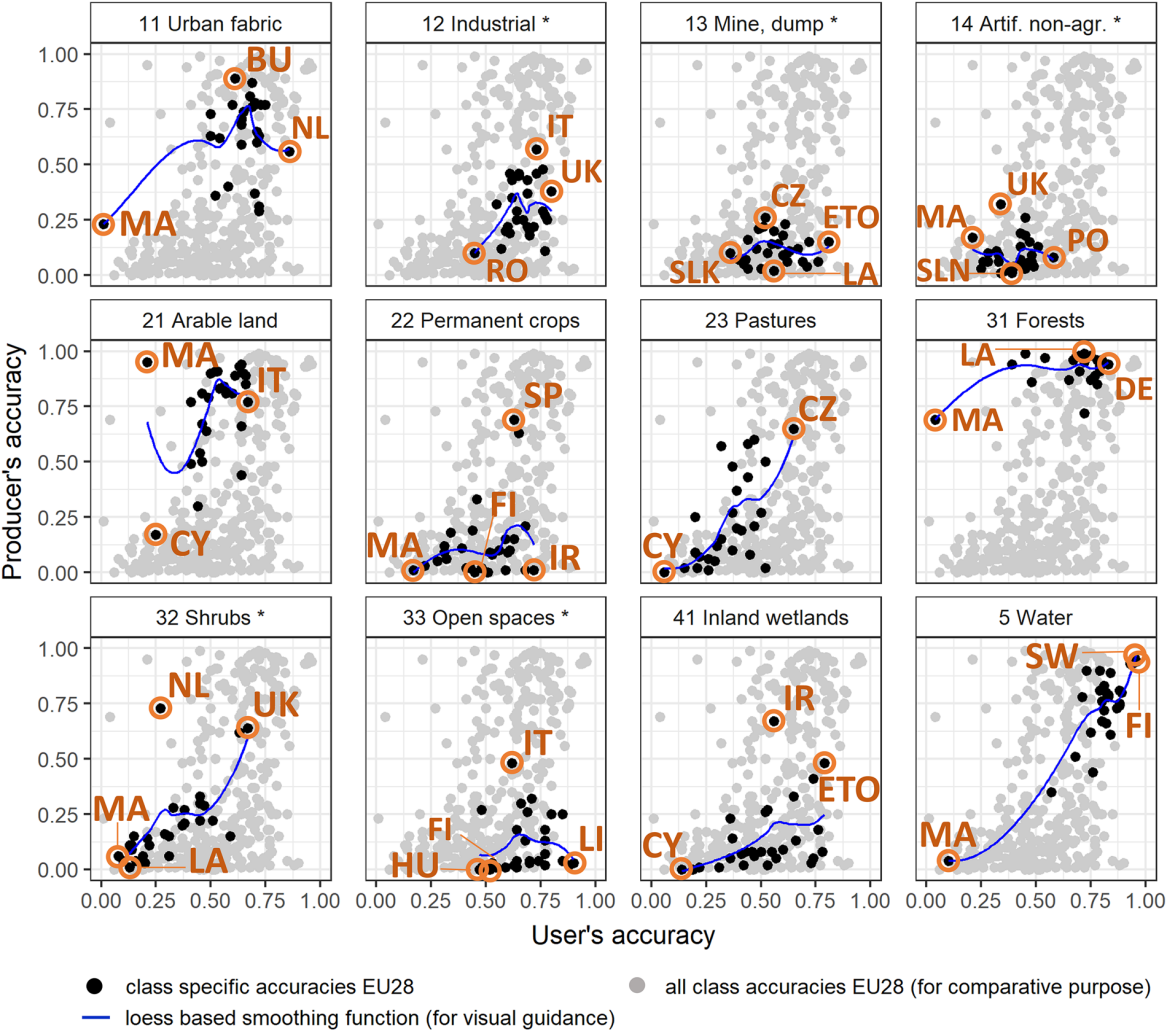
Area cross tabulations of existing labels (reference) and the respective classification result (map); producer- and user accuracy were used to express their spatial conflation; *full class names are provided in the second column of Table 1; orange labels are EU country codes for highlighting extremes.

The facets shown in Fig. 2 highlight various areas of the study site and highlight the properties of our product across the various landscapes of the EU. Figure 2 facet a) shows Utrecht province within the Netherlands, a fine grained spatial explicit detailed depictions of the water body, pastures, arable land, and residential areas. This area was almost entirely covered by OSM contributions and therefore did require only minor deep learning prediction. The proportion of available suitable OSM data relative to its total area for the Netherlands (NL) was 96.4% (Fig. 3) and therefore is almost completely available at fine <1 m resolution, the spatial detail was dependent on the availability of OSM data. Only Romanian was more extensively covered by original OSM data. The level of detail depicted particularly for countries with major OSM coverage cannot be achieved by the sole use of remote sensing. Facet e) within Fig. 2 highlighted a wetland river estuary in fine spatial detail close to Bordeaux. Due to their diverse set of spectral properties and dynamic behaviour wetlands can be difficult to pick up using remote sensing only^34^ using existing OSM contributions can be an option to bypass this challenge, as demonstrated here. Facet d) and f) showed the Po valley in Italy and the lake Balaton in Hungary respectively. Both examples demonstrate the products sensitivity to distinguish semi-natural areas and arable land at large scale. Facets b) and c) contain pixelated elements that were created because of the deep learning classification completing the OSM data, the loss of spatial detail was particularly evident for facet c) where the 10 m spatial resolution of the predicted LULC fails to accommodate a clean distinction of the river crossing the city and the numerous bridges crossing the river. Facet b) provided a representative example of the overall characteristics found in OSMlanduse, fine grain resolution of vector geometries mixed with 10 m classified pixels blocks.

The variation of resolution and data quality was dependent on the availability of OSM data. Figure 3 showed that most EU countries had major LULC relevant direct OSM coverage. Most striking examples where RO, NL, CZ, SI with more than 90% directly available OSM data and IT, BG, GR, ES, IE and PT with less than 40% available OSM data. There is no specific spatial pattern regarding the availability of OSM data yet mapping activity can be depended on the regional OSM communities^26^.

## Data Availability

The scripts used in this research are licensed under the GNU General Public License 3.0 and written in Python, with some routines calling SQL and available here https://github.com/schultzheidelberg/OpenStreetMap-land-use-for-Europe-2020. Resources provided include land use to shape for extracting training polygons from OpenStreetMap, links to successful models for five countries, and processing routines, including our high-performance cluster implementations. Pre-Processing and procurement of remote sensing data collection of Sentinel 2 best pixel median composite was conducted via generic Food and Agriculture Organization www.sepal.io platform.
